# Identification of a novel gene signature predicting response to first-line chemotherapy in BRCA wild-type high-grade serous ovarian cancer patients

**DOI:** 10.1186/s13046-022-02265-w

**Published:** 2022-02-04

**Authors:** Marianna Buttarelli, Alessandra Ciucci, Fernando Palluzzi, Giuseppina Raspaglio, Claudia Marchetti, Emanuele Perrone, Angelo Minucci, Luciano Giacò, Anna Fagotti, Giovanni Scambia, Daniela Gallo

**Affiliations:** 1 Unità di Medicina Traslazionale per la Salute della Donna e del Bambino, Dipartimento Scienze della Salute della Donna, del Bambino e di Sanità Pubblica, Fondazione Policlinico Universitario A. Gemelli, IRCCS, Largo A. Gemelli 8, 00168 Rome, Italy; 2 Dipartimento Universitario Scienze della Vita e Sanità pubblica – Sezione di Ginecologia ed Ostetricia - Università Cattolica del Sacro Cuore, Largo A. Gemelli 8, 00168 Rome, Italy; 3 Bioinformatics Facility Core Research, Gemelli Science and Technology Park (GSTeP) Fondazione Policlinico Universitario A. Gemelli, IRCCS, Roma, Italy; 4 Dipartimento Scienze della Salute della Donna, del Bambino e di Sanità Pubblica, Fondazione Policlinico Universitario A. Gemelli, IRCCS, Roma, Italy; 5 Molecular and Genomic Diagnostics Unit (MGDUnit), Fondazione Policlinico Universitario A. Gemelli IRCCS, Roma, Italy

**Keywords:** HGSOC, Drug-resistance, Patient stratification, Transcriptomic, Biomarkers, Bioinformatics, Random forest classifier model, Primary ovarian cancer cells

## Abstract

**Background:**

High-grade serous ovarian cancer (HGSOC) has poor survival rates due to a combination of diagnosis at advanced stage and disease recurrence as a result of chemotherapy resistance. In BRCA1 (Breast Cancer gene 1) - or BRCA2-wild type (BRCAwt) HGSOC patients, resistance and progressive disease occur earlier and more often than in mutated BRCA. Identification of biomarkers helpful in predicting response to first-line chemotherapy is a challenge to improve BRCAwt HGSOC management.

**Methods:**

To identify a gene signature that can predict response to first-line chemotherapy, pre-treatment tumor biopsies from a restricted cohort of BRCAwt HGSOC patients were profiled by RNA sequencing (RNA-Seq) technology. Patients were sub-grouped according to platinum-free interval (PFI), into sensitive (PFI > 12 months) and resistant (PFI < 6 months). The gene panel identified by RNA-seq analysis was then tested by high-throughput quantitative real-time PCR (HT RT-qPCR) in a validation cohort, and statistical/bioinformatic methods were used to identify eligible markers and to explore the relevant pathway/gene network enrichments of the identified gene set. Finally, a panel of primary HGSOC cell lines was exploited to uncover cell-autonomous mechanisms of resistance.

**Results:**

RNA-seq identified a 42-gene panel discriminating sensitive and resistant BRCAwt HGSOC patients and pathway analysis pointed to the immune system as a possible driver of chemotherapy response. From the extended cohort analysis of the 42 DEGs (differentially expressed genes), a statistical approach combined with the random forest classifier model generated a ten-gene signature predictive of response to first-line chemotherapy. The ten-gene signature included: CKB (Creatine kinase B), CTNNBL1 (Catenin, beta like 1), GNG11 (G protein subunit gamma 11), IGFBP7 (Insulin-like growth factor-binding protein 7), PLCG2 (Phospholipase C, gamma 2), RNF24 (Ring finger protein 24), SLC15A3 (Solute carrier family 15 member 3), TSPAN31 (Tetraspanin 31), TTI1 (TELO2 interacting protein 1) and UQCC1 (Ubiquinol-cytochrome c reductase complex assembly factor). Cytotoxicity assays, combined with gene-expression analysis in primary HGSOC cell lines, allowed to define CTNNBL1, RNF24, and TTI1 as cell-autonomous contributors to tumor resistance.

**Conclusions:**

Using machine-learning techniques we have identified a gene signature that could predict response to first-line chemotherapy in BRCAwt HGSOC patients, providing a useful tool towards personalized treatment modalities.

**Supplementary Information:**

The online version contains supplementary material available at 10.1186/s13046-022-02265-w.

## Background

Ovarian cancer is the second most common cause of gynecologic cancer death in women worldwide. Nearly 314,000 women were estimated to have been diagnosed with ovarian cancer and 207,000 to have died from disease in 2020, with rates varying across the world [1]. Over 90% of ovarian malignancies are categorized as epithelial ovarian cancers and HGSOC is estimated to be 50–60% of all ovarian malignancies [2]. HGSOC classically presents at advanced stage, and cytoreductive surgery and systemic platinum-taxane combination chemotherapy represent the standard of care for patients. However, most tumors eventually develop drug resistance, with a 5-year survival generally below 30% [3]. Chemosensitivity to platinum-based chemotherapy is a prognostic factor for patient survival and a significant determinant for subsequent treatment, including maintenance therapy with PARP inhibitors (PARPI). Indeed, the use of PARPI has markedly transformed the therapeutic armamentarium in HGSOC over the last 5 years, since women with HRD (homologous recombination deficiency), in particular BRCA1/2-mutated HGSOCs, greatly benefit from PARPI treatment [4]. Actually, PARPI approval has been granted for patients with ovarian cancer, both in the front line setting and as maintenance or treatment in recurrent platinum-sensitive disease regardless of HRD status [4, 5].

Notably, resistance (and progressive disease) occurs more often in BRCA1- or BRCA2wt than in mutated BRCA HGSOC patients. In platinum-resistant OC, the choice of therapy is challenging and its failure is eventually responsible for almost all deaths from this cancer [6]. Accordingly, advanced-stage, BRCA1/2wt HGSOC patients have a worse prognosis, with shorter PFS (progression free survival) than those with BRCA mutations [7]. In the era of personalized cancer therapy, validated predictive biomarkers do not exist for platinum sensitivity in women with BRCAwt ovarian cancers and predicting platinum sensitivity remains a challenge [8]. Despite various approaches have been developed, including mutational signature, transcriptomic signatures, tumor mutational burden, and functional biomarkers, no definitive results have been reached yet to guide precision treatment for women with BRCAwt ovarian cancers [8, 9]. Actually, the elevated level of intra- and inter-tumor and inter-patient heterogeneity that characterized HGSOC has been shown to be present even prior to exposure to selective pressures of therapy, this having significantly hampered biomarker discovery into actionable clinical parameters [8].

The present study aimed at the identification of a biomarker signature that could predict response to first-line therapy in BRCAwt HGSOC patients. Patient phenotyping would allow patient allocation to personalized treatment procedures, with significant benefits to both patients and healthcare system.

## Materials and methods

### Study population

This was a retrospective study including 44 women admitted to the Gynecologic Oncology Unit, Fondazione Policlinico Universitario A. Gemelli IRCCS, Roma, from Apr 2014 to Sep 2018. Eligible patients were defined as having histologically confirmed diagnosis of HGSOC, BRCAwt status and pretreatment frozen biopsy available at XBIOGem-Biorep Gemelli Biobank. Pathology-based estimates of the percentage of tumor cells were evaluated from hematoxylin-eosin-stained slides. Samples included in the analysis had tumor purity higher than 70% (Fig. S1). Ethical approval for the study was obtained from Ethics Committee of Fondazione Policlinico Universitario A. Gemelli IRCCS, Roma (Prot. 2552/20 ID 2694).

Patients were treated either with primary cytoreductive surgery followed by 6 conventional cycles of chemotherapy (PDS) or with 3 or 4 cycles of neoadjuvant chemotherapy before surgery and 3 postoperative chemotherapy cycles (NACT + IDS). Patients received platinum-based chemotherapy; introduction of bevacizumab in combination with standard chemotherapy and as maintenance therapy was allowed following its approval in Italy (January, 2014). Response to chemotherapy and progression were defined according to Response Evaluation Criteria in Solid Tumors (RECIST) and Gynecologic Cancer Intergroup (GCIG) criteria [10, 11]. To define chemosensitivity, the common definition of platinum resistance was used, identifying as “resistant” (PFI < 6 months) patients who relapsed less than 6 months after chemotherapy was stopped, or that progressed during therapy, and as “sensitive”, patients relapsing 6 months or more after chemotherapy [6]. In order to maximize the identification of potential differences in the biomarker profile associated with chemotherapy responsiveness, we decided to focus our analysis on patients with PFI < 6 months (R, resistant) and PFI > 12 months (S, sensitive).

Selected patients were divided in two cohorts. Targeted RNA sequencing was performed on samples from the discovery cohort, including 14 BRCAwt patients (i.e. 7S and 7R). This analysis allowed the identification of a restricted panel of 42 DEGs that could discriminate chemotherapy-sensitive from -resistant patients. This gene set was tested by HT RT-qPCR in an extended cohort including 25S and 19R patients (validation cohort). Patients’ characteristics are reported in Table 1.

**Table 1:** Clinicopathological characteristics of HGSOC patient cohort

Characteristics	Discovery cohort		Validation cohort	
	Sensitive No. (%)	Resistant No. (%)	Sensitive No. (%)	Resistant No. (%)
All cases	7	7	25	19
Median Age, years (range)	55 (48–65)	62 (47–71)	59 (37–73)	59 (32–79)
FIGO Stage				
III	6 (85.7)	6 (85.7)	22 (88.0)	14 (73.7)
IV	1 (14.3)	1 (14.3)	3 (12.0)	5 (26.3)
Primary treatment strategy				
PDS	7 (100)	6 (85.7)	20 (80.0)	8 (42.1)
NACT+IDS	–	–	5 (20.0)	9 (47.4)
NACT	–	1 (14.3)	–	2 (10.5)
Primary chemotherapy				
Platinum/Paclitaxel	2 (28.6)	4 (57.1)	11 (44.0)	11 (57.9)
Platinum/Paclitaxel/Bevacizumab	5 (71.4)	3 (42.9)	14 (56.0)	8 (42.1)
Residual tumor at surgery				
RT = 0	5 (71.4)	4 (57.1)	17 (68.0)	10 (52.6)
RT > 0	1 (14.3)	2 (28.6)	5 (20.0)	5 (26.3)
Not available/applicable	1 (14.3)	1 (14.3)	3 (12.0)	4 (21.1)

*HGSOC* high-grade serous ovarian cancer, *PDS* primary debulking surgery, *NACT* Neoadjuvant chemotherapy, *IDS* interval debulking surgery

### Generation of primary cancer cells from patient tissues

This study included 7 patients with newly diagnosed, histologically confirmed BRCAwt HGSOCs, admitted to the Gynaecologic Oncology Unit, Fondazione Policlinico Universitario A. Gemelli, IRCCS, Roma, between May 2020 and February 2021. The study was approved by the local Ethics Committee and Institutional Review Board (Protocol 19402/18 ID: 2045). All data were managed using anonymous numerical codes (OV.GEM). Patients’ characteristics are reported in Table S1.

### Primary cancer cell culture and characterization

Samples for cell cultures were collected at surgery from “left over tissues” by using sterile scalpels. Tumour specimens were placed into sterile tubes containing culture medium [DMEM (Dulbeccoʼs modified Eagle′s medium) supplemented with 1% kanamycin] pre-warmed to 37 °C. Thereafter, tissue samples placed in 60 mm Petri dishes containing PBS (Phosphate-buffered saline) with 10% fetal bovine serum (FBS), were finely minced by surgical blades into small fragments.

Tumour tissues were enzymatically digested using the Tumor Dissociation kit (Miltenyi Biotec Bologna Italy). According to manufacturer’s protocol, fragments were incubated in 5 ml DMEM medium containing enzymes mix for 30 min at 37 °C. The cell suspension was applied to a MACS SmartStrainer (70 μm), placed on a 50 mL tube and washed with 20 ml of PBS with 10% FBS, centrifuged and resuspended in complete DMEM/F12 (1:1) supplemented with heat inactivated foetal bovine serum (10%), glutamine (2 mM) and kanamycin (2 mM) (Life Technologies, CA, USA). Cells were maintained at 37 °C in the presence of 5% CO_2_ and 95% humidified air. Medium was changed 48/72 h after initial plating and every 3 days thereafter. Stromal cells, primarily fibroblasts, progressively disappeared during the subcultivation process. Each patient-derived cell line was characterized by morphology, immunocytochemistry, and immunofluorescence.

Morphological features were studied using an inverted microscope DM IL LED (Leica Microsystems, Milan, Italy). Cells cultures were then characterized using specific epithelial and stromal markers. To this end, cells were centrifuged at 600 rpm for 5 min at cytospin centrifuge, cytospins were fixed with 4% paraformaldehyde for 20 min at RT and then permeabilized in 0.5% v/v Triton X-100 in PBS for 10 min. The endogenous peroxidase was blocked with 3% H_2_O_2_ for 5 min. After washing twice with PBS, cells were incubated with a blocking solution containing 20% normal horse serum in PBS for 30 min at RT. After incubation with the primary antibodies, i.e. anti-cytokeratin 7 (Clone OV-TL 12/30, Agilent Dako, Santa Clara, CA USA, ready-to-use) and anti-human fibroblasts (clone TE-7, Sigma Aldrich, Merck KGaA, Darmstadt, Germany dilution 1:100), for 1 h at RT in a humidified chamber, samples were rinsed with PBS and incubated with the secondary antibody, EnVision System-HRP (Dako), for 30 min at RT. Immunoreactivity was detected using the 3,3′-diaminobenzidine substrate (DAB substrate System, Agilent Dako). Slides were counterstained with Mayer’s Haematoxylin, dehydrated in ethanol and xylene, and finally mounted.

Finally, expression of cytokeratin 7 was also confirmed by fluorescence microscopy. To this end, cells were seeded in 6-well plates containing coverslip in complete growth medium, fixed in 4% paraformaldehyde for 20 min at 20 °C, and permeabilized in 0.5% v/v Triton X-100 in PBS for 10 min, prior to be blocked with 5% v/v goat serum and 0.1% v/v Triton X-100 in PBS for 1 h. Immunofluorescence staining was obtained using anti- anti-cytokeratin 7 (Clone OV-TL 12/30, Agilent Dako, ready-to-use) following overnight incubation at 4 °C. After washing, cells were incubated with secondary antibody anti-mouse Alexa Fluor-488 conjugate (1:200) (Thermo Fisher Scientific Waltham, MA), in the dark for 30 min at 20 °C. Coverslip was mounted onto slides using an antifade mounting reagent containing DAPI. Slides were observed under a fluorescence microscope (Leica Biosystems, Newcastle, UK) using a 100X oil immersion objective.

### Established HGSOC cell lines

The human ovarian carcinoma cell line COV318 was obtained from the European Collection of Cell Cultures (ECACC, Salisbury, UK), while NIH:OVCAR-3 and OV-90 from the American Type Culture Collection (ATCC, Milan, Italy). Susan Horwitz (Albert Einstein Medical College) donated the HEY cell line. COV318 is a human ovarian epithelial-serous carcinoma cell line established from a peritoneal ascites [12]. The NIH:OVCAR-3 line was established from the malignant ascites of a chemotherapy-treated patient with progressive adenocarcinoma of the ovary [13]. OV-90 cells were derived from a chemotherapy-naive grade 3, stage IIIC, malignant papillary serous adenocarcinoma [14]. HEY cell line was derived from a human ovarian cancer xenograft originally grown from a peritoneal deposit of a patient with moderately differentiated papillary cystadenocarcinoma of the ovary [15].

NIH:OVCAR-3 and HEY cells were cultured in RPMI 1640 (Roswell Park Memorial Institute Medium) and COV318 in DMEM (Sigma Aldrich). OV-90 were cultured in 1:1 mixture of MCDB 105 medium (containing a final concentration of 1.5 g/L sodium bicarbonate) and Medium 199 (containing a final concentration of 2.2 g/L sodium bicarbonate). Medium was supplemented with 10% FBS for COV318, NIH:OVCAR-3 and HEY, and 15% FBS for OV-90, plus MEM (Minimum Essential Medium) Non-Essential Amino Acid 1%, glutamine 1 mM and kanamycin 1%. Cells were grown in a fully humidified atmosphere of 5% CO2/95% air, at 37 °C. Cells were routinely tested for mycoplasma (MycoAlert mycoplasma detection kit, LONZA, Rockland, ME, USA) and validated by STR (Short Tandem Repeat) DNA profiling (BMR Genomics srl, Padua, Italy).

### Cytotoxicity assay

Cells were seeded in 96-well plate overnight at a density of 5 × 10^3^ per well, and then treated with different concentrations (0.001 to 100 μM) of Paclitaxel or Cisplatin (both from Sigma Aldrich) for 72 h. After incubation, 10 μL Cell Counting Kit-8 (Sigma Aldrich) was added into each well for 1 h. The OD (optical density) of each sample was detected at 450 nm using a microplate reader (Enspire, Perkin Elmer, Walthman, MA, USA). Each treatment was performed in triplicate. The mean optical density in the indicated concentrations was used to calculate the percentage of cell viability.

### Isolation of RNA

Total RNA was isolated from HGSOC tumors and primary cells using the AllPrep DNA/RNA/Protein Mini Kit (Qiagen, Milan, Italy) according to the manufacturer’s protocol and stored at − 80 °C until analyzed. Recovered RNA concentration and quality were measured using the Nanodrop (Thermo Fisher Scientific) and Agilent 2100 Bioanalyzer (Agilent Technologies), respectively.

### RNA-seq analysis

A targeted RNA sequencing for gene expression and transcriptome analysis was performed using the Ion Ampliseq Transcriptome Human Gene Expression kit (Thermo Fisher Scientific) on Ion S5™ System (Thermo Fisher Scientific), on a discovery cohort (i.e. 7S and 7R BRCAwt HGSOC patients). This kit has been designed to profile over 20,000 distinct human RNA targets using a highly multiplexed amplification method. Briefly, cDNA was first generated using the SuperScript® VILO™ cDNA Synthesis kit from 10 ng of total RNA obtained from tumor samples. Library preparation was carried out using an Ion Chef System (Thermo Fisher Scientific) according to the manufacturer’s instructions. Briefly, barcoded libraries were generated from cDNA using an Ion AmpliSeq Chef Solutions DL8 Kit (Thermo Fisher Scientific) and an Ion Ampliseq Transcriptome Human Gene Expression kit (Thermo Fisher Scientific). The Ion AmpliSeq™ technology accurately maintains expression levels of all targeted genes as previously described [16]. Amplified cDNA Libraries were quantified using the Qubit™ 3.0 Fluorometer (Thermo Fisher Scientific). Libraries were then diluted to 100pM and pooled equally, with eight individual samples per pool. Pooled libraries were amplified using emulsion PCR on Ion Chef and enriched following the manufacturer’s instructions. Template libraries were then sequenced on an Ion S5™ sequencing system, using Ion 540 Chip and an Ion 540 kit–Chef Kit (ThermoFisher Scientific).

### Assessment of mRNA expression profiles using Fluidigm 48.48 dynamic arrays

The 42-gene panel identified by RNA-seq analysis was tested by HT RT-qPCR in an extended cohort including 25S and 19R patients (validation cohort). Analysis also included mRNAs isolated from primary HGSOC cells. Total RNA was tested using 48.48 dynamic array (Fluidigm Corporation, San Francisco, CA, USA) and a Biomark platform, following the manufacturer’s protocol, as previously described [17]. The list of 42 genes evaluated in the study, along with primers used, is shown in Table S2. The geometric mean of RPL13A, RPL4 and GAPDH was taken as reference genes, following GeNorm and Normfinder algorithm [18, 19]. Data were analyzed as dCt values (Ct_target_-Ct_gmeanref_) according to 2^-ΔΔCt^ method [20].

### RNA-seq data analysis

Primary analysis for AmpliSeq sequencing data of all samples was performed using the ampliSeqRNA plugin available for Ion Torrent Software Suite version 5.10.1. Raw reads were mapped to a custom reference sequence set (hg19 AmpliSeq Transcriptome v1.1) using the Torrent Mapping Alignment Program (TMAP) [16]. Raw read counts of the targeted genes were carried out using samtool (samtools view –c –F 4 –L bed_file bam_file), as part of ampliSeqRNA plugin. Differential expression analysis was performed using the DEBrowser R package [21], filtering out genes showing average raw counts less than 50. Batch effect correction was applied over samples coming from different runs, using the ComBat algorithm (the original algorithm is implemented in the sva R pakage [22]). The DEseq2 algorithm [23] was then used to determine DEGs at a significance threshold of Benjamini-Hochberg (BH) adjusted *P* value < 0.05 [24]. Fold changes (FC) are reported as resistant over sensitive expression values. A further stringency threshold of BH-adjusted *P*-value <5e-03 and absolute log_2_FC > 1 lead to the selection of 42 top DEGs, used for further validation through HT RT-qPCR.

### RNA-seq/HT RT-qPCR correlation

Expression data reproducibility was assessed by correlating standardized RNA-seq raw counts with HT RT-qPCR standardized -ΔCt values. Since standardization does not affect correlation, the accordance between the two techniques was tested with a simple linear regression, using the lm function of the R base environment [25]. The estimated slope of the regression line was interpreted as Pearson correlation coefficient, and a *P* value < 0.05 was interpreted as a significant correlation between RNA-seq and HT RT-qPCR values. Separate regressions were done for S and R subjects, to highlight possible differences due to the response to the treatment.

### Differential expression evaluation on HT RT-qPCR data

High-throughput RT-qPCR -ΔCt values of the 42 selected DEGs, from 25S and 19R subjects, were compared using a Wilcoxon rank sum test (two-sided), implemented within the R base environment. The effect size was the estimated shift between negative ΔCt values in R versus S subjects, assuming a significance level of 0.05.

### Random Forest Classifier (RFC) for prediction

To validate the significant DEGs found through the Wilcoxon rank sum test, a 4-fold cross-validation (CV) RFC was set to predict sensitive and resistant phenotypes, and to provide a rank of DEGs contribution to the classification process. During the training procedure, mean decrease accuracy (MDA), mean decrease Gini (MDG) impurity and Brier scores were computed. Brier scores were calculated for each subject and used as a measure of classification uncertainty. Subjects with a Brier score greater than 1 were discarded to improve both prediction accuracy and variable ranking. After removing high-uncertainty subjects, a new training process was started, and MDA and MDG were calculated, measuring the contribution that each variable has in the prediction accuracy and in the entropy of the classification process, respectively. Since MDA and MDG have different scales, their values were normalized from 0 to 100, where the worst predictor (gene) scores 0 and the best one scores 100. The ranking score for each gene is then the average of the percent MDA and MDG values.

The overall performance of the classification procedure was described in terms of Sensitivity, Specificity, Precision (or Positive Predictive Value, PPV), F1 score, Accuracy, False Positive rate (FPR), False Discovery Rate (FDR), and False Negative Rate (FNR). The method was implemented using the R packages randomForest [26] and CMA [27], for RFC building and Brier score calculation, respectively.

### RICF-based heuristic analysis for primary cell lines validation

Gene deregulation trends were also validated on RT-qPCR data from primary cell lines (OV.GEM). The analysis was done separately upon two different treatments: Paclitaxel and Cisplatin. For each treatment, experimental data were used to establish sensitivity or resistance (outcome). Given the small number of statistical units (7 OV.GEM lines), a Residual Iterative Conditional Fitting (RICF)-based heuristic was applied to detect genes with significant differences in resistant versus sensitive cell lines. The RICF method [28] achieves least squares convergence to exact maximum likelihood estimation of model parameters, in case of high dimensionality. Furthermore, the heuristic disables standard error estimation, and generates *P* values by fast randomization of group labels (resistant, sensitive). The entire procedure is implemented in the SEMrun() function of R package SEMgraph [29]. A significance level of 0.05 was set to determine gene regulation.

### Over Representation Analysis (ORA)

To functionally annotate the 42 DEGs, we performed an ORA over the Kyoto Encyclopedia of Genes and Genomes (KEGG) database, using the Enrichr web-based tool [30]. Pathway enrichment was evaluated according to 3 criteria: (i) absolute number of genes from the input set belonging to a given pathway, (ii) two-sided Fisher’s exact test Odds Ratio, 95% confidence intervals and *P*-value (including Benjamini-Hochberg adjusted P-value), to evaluate the association between the input gene set and the given pathway, (iii) a combined score derived from the product of the -log10 of the Fisher’s exact test P-value and the z-score transformation of the Fisher statistic.

### Figure creation

Unless stated differently, pictures have been generated using the R package ggplot2 [31].

### Analysis of publicly available datasets

In silico prediction of the possible genes association network for the 42 DEGs was performed using the GeneMANIA plugin in Cytoscape v.3.8.2, an integrated interaction network program that predicts gene functions and possible interaction networks using many large publicly available datasets. Association data include protein and genetic interactions, pathways, co-expression, co-localization and protein domain similarity. For the estimation of the networks weight, the default network weighting method was chosen (Assigned based on query gene). This method maximize connectivity between all input genes. The GeneMANIA plugin in Cytoscape v.3.8.2 was used to generate the interaction networks [32].

cBioPortal (www.cbioportal.org) was used to access the publicly available Ovarian Serous Cystadenocarcinoma (TCGA, PanCancer Atlas) dataset and queried for co-expression of UQCC1 and CDR2 (Cerebellar Degeneration Related Protein 2), with setting using database default.

### Statistics

Means and standard deviation (SD) were calculated for all data points, from at least two independent experiments. Dose-response curve-fit was calculated with v6.0 GraphPad Prism (GraphPad software, La Jolla, CA, USA).

## Results

### Identification of differentially expressed genes between sensitive and resistant HGSOC patients

To identify specific gene markers of therapy response, we compared pretreatment biopsies from 7 sensitive (S, PFI > 12 months) and 7 resistant (R, PFI < 6 months) patients, using a targeted whole transcriptome sequencing approach.

**Fig. 1:**
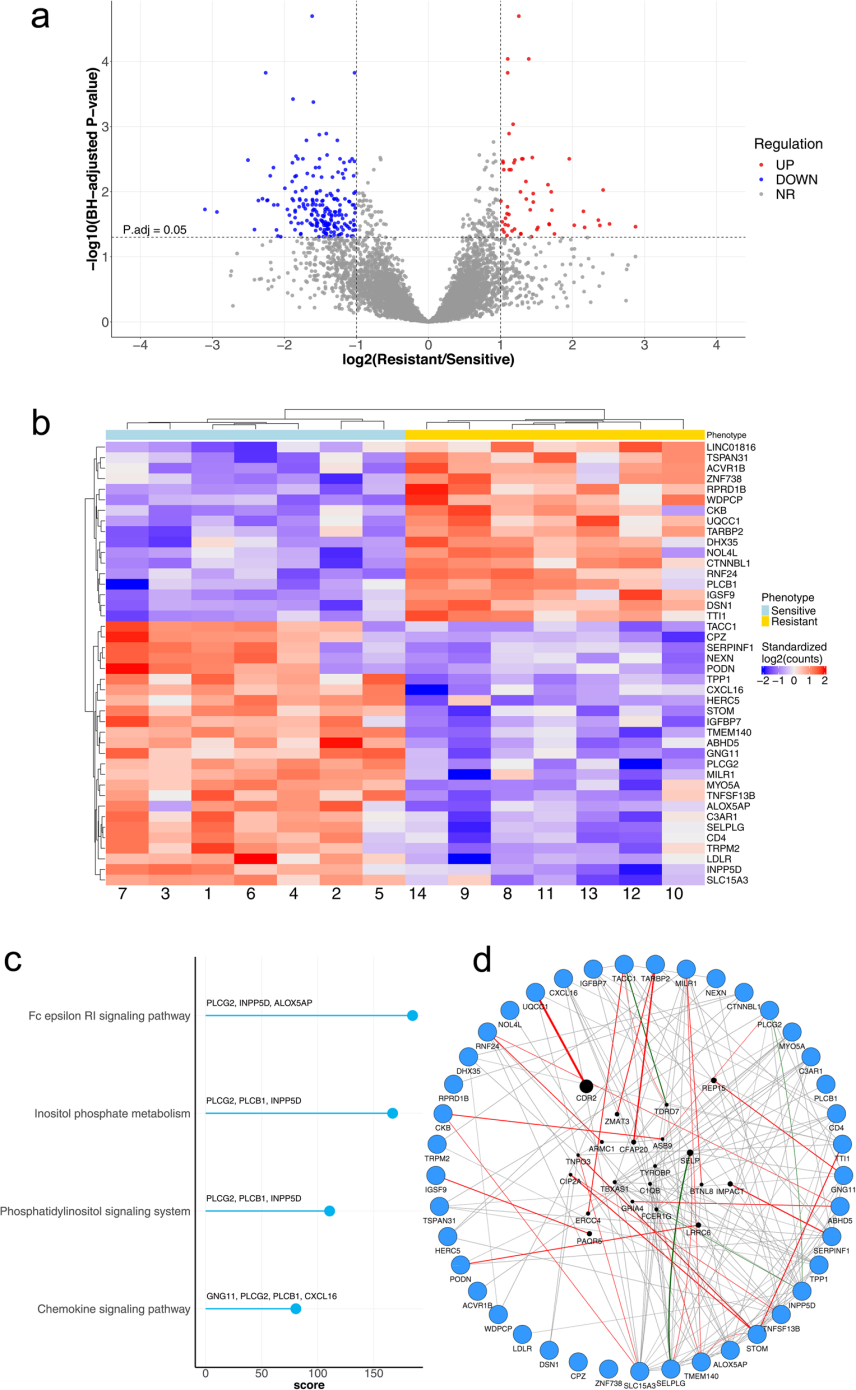
**a** Volcano plot of the RNA-seq data from high grade serous ovarian cancer (HGSOC) patients (*n* = 14, 7 sensitive and 7 resistant). The plot reports the negative log_10_ of the Benjamini-Hochberg adjusted *P*-value against the log_2_ fold-change (resistant vs sensitive) of the RNA-seq expression counts. The significance level for gene regulation is set to 0.05. Each gene is a dot, highlighted in blue for down-regulated genes, red for up-regulated, and grey for not-regulated ones. **b** Hierarchical clustering of the top 42 DEGs (Differentially expressed genes). Hierarchical agglomerative clustering based on Euclidean distance of resistant and sensitive subjects, calculated on RNA-seq standardized raw counts, over the top-42 DEGs (RNA-seq BH-adjusted *P*-value <5E-03 and |log_2_FC| > 1). **c** Barplot of the KEGG enrichment analysis. Barplot showing the enrichment score (x axis) and supporting genes (labels over the bars) of the 4 enriched KEGG pathways among the 42 RNA-seq top-DEGs. Enrichment data was extracted from the output of the online tool Enrichr. **d** GeneMania interaction networks for the 42 DEGs. The network was spatially represented using the Cytoscape degree sorted circle layout, in which all nodes with the same numbers of links are located together around the circle. The circle is composed of the 42 query genes (except the lncRNA LOC100133985) found in our study, whereas the genes inside are the result genes identified by GeneMANIA. The colour of the line connecting the genes indicates the type of communication. Our diagram encompasses co-expression in grey lines, physical interactions in red lines, pathway in green lines. The thicknesses of the links (or edges) between the genes are proportional to the interaction strengths, whereas the node size for each gene is proportional to the rank of the gene based on its importance (or gene score) within the network

A total of 10,807 genes were found to be measurably expressed (raw read counts > 50) in tumor samples (Table S3). Under a threshold of adjusted *P* value (padj) < 0.05 and |log2FC| > 1, we identified 258 DEGs between S and R patients, including 55 upregulated and 203 downregulated genes (Fig. 1a, red and blue colored dots). After applying a more stringent filtering approach (padj< 0.005, |log2FC| > 1), 42 DEGs were found to be able to discriminate between the two different phenotypes, as shown by heatmap of sample-to-sample distance (Fig. 1b). Among the 42 DEGs, 17 were upregulated and 25 downregulated in R compared to the S patients. Magnitude of fold changes (log2 scale) was within − 2.51 to + 1.95. Fold changes and molecular functions of the 42 DEGs are reported in Table S4.

### Pathway enrichment analysis

To obtain further insight into the function of DEGs, they were uploaded to the KEGG database. As shown in Fig. 1c, KEGG pathway analysis found 4 significantly enriched pathways: Fc Epsilon RI signaling pathway (combined score 184.64; padj 0.027), Inositol phosphate metabolism (combined score 166.85; padj 0.027), Phosphatidylinositol signaling system (combined score 110.64; padj 0.033) and Chemokine signaling pathway (combined score 80.53; padj 0.027), with the following genes involved: ALOX5AP (Arachidonate 5-lipoxygenase-activating protein), CXCL16 (C-X-C motif chemokine ligand 16), GNG11, INPP5D (Inositol Polyphosphate-5-Phosphatase D), PLCB1 (Phospholipase C beta 1), and PLCG2.

### Gene-gene functional interaction network construction

We also used GeneMANIA plugin in Cytoscape v.3.8.2 to analyze the 42 DEGs with functionally similar genes together, to obtain regulatory networks. According to the annotation information recorded in GeneMANIA, a network involving the 42 DEGs and 20 related genes was established (Fig. 1d). Only physical interaction, pathway and co-expression were included in the network visualization. Notably, a strong physical interaction was evident between UQCC1 and CDR2 (Cerebellar degeneration-related protein 2) (Fig. 1d). We focused our attention on this finding, since CDR2 (normally present in cerebellar Purkinje neurons and brainstem neurons and testes) is a tumor antigen ectopically expressed in more than 50% of ovarian tumors [33]. We therefore sought to determine whether any evidence exists for the relationship between UQCC1 and CDR2 in serous ovarian cancer. Analyzing The Cancer Genome Atlas (TCGA) data via cBioPortal, we found that UQCC1 expression significantly (*P* < 0.05) inversely correlated with CDR2.

### Validation of RNA-seq data by HT RT-qPCR

**Fig. 2:**
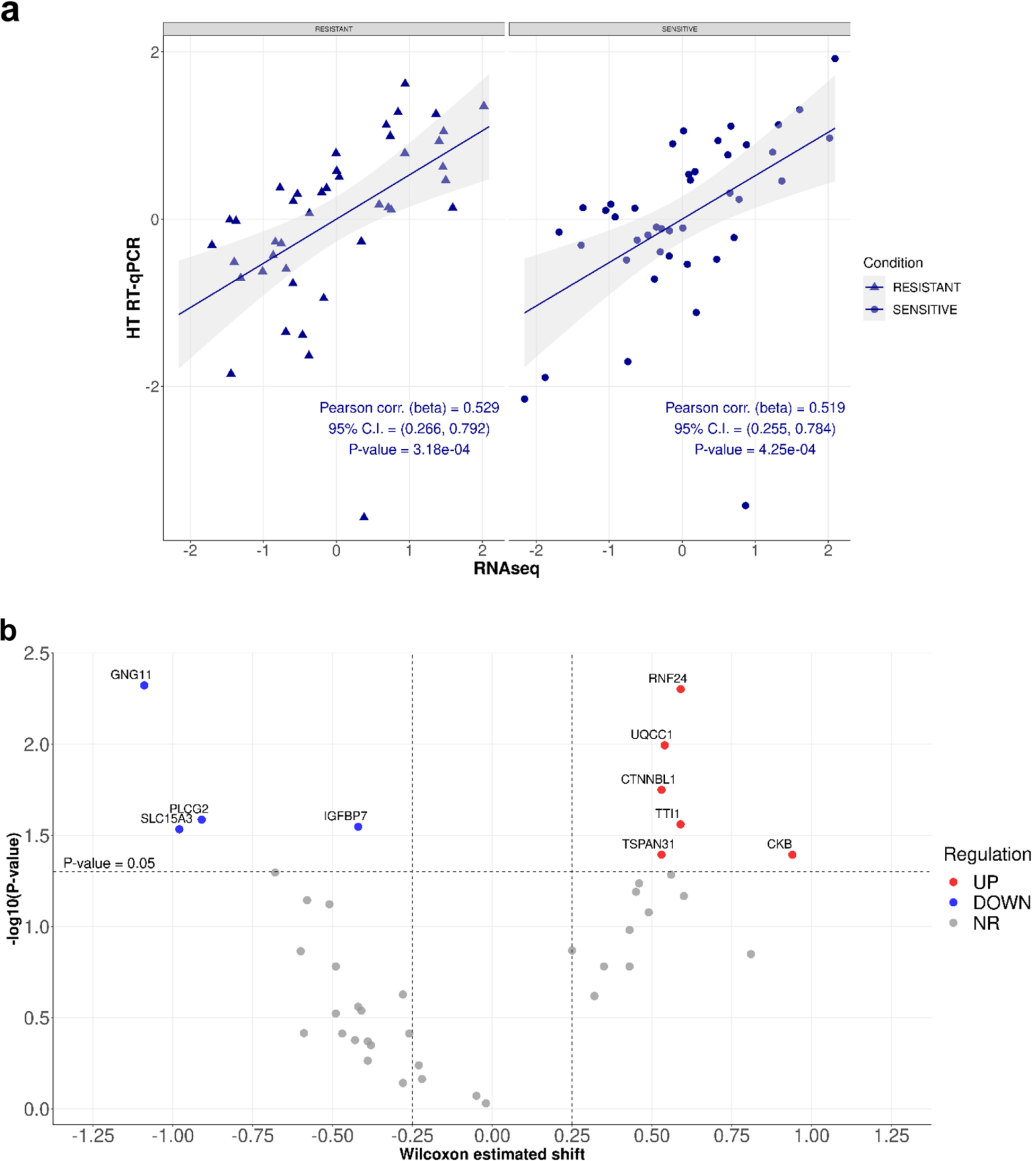
**a** High-throughput RT-qPCR/RNA-seq correlation over the top-42 DEGs. Simple linear regression (95% confidence interval in grey) was performed for resistant (triangles) and sensitive (circles) subjects, respectively. Pearson correlation is estimated as regression curve slope. Each regression also reports 95% confidence intervals of the estimates (95% CI) and corresponding *P*-values. **b** Volcano plot of the HT RT-qPCR data. The plot reports the negative log10 of the Benjamini-Hochberg adjusted *P* value against the log2 fold-change (resistant vs. sensitive). The significance level for gene regulation is set to 0.05. Each gene is a dot, highlighted in blue for down-regulated genes, red for up-regulated, and grey for not-regulated ones

The 42 DEGs panel identified by RNA-seq was validated, by HT RT-qPCR, in an extended cohort of 25S and 19R patients (Validation cohort). Notably, analysis based on the Pearson’s correlation coefficient indicated the positive correlation in gene expression measurements between RNA-seq and HT RT-qPCR (Fig. 2a), in both conditions (i.e., S and R patients).

Differentially expressed genes were identified via volcano plot, and genes with a *P* value less than 0.05 were filtered, this allowing the identification of ten genes significantly associated with chemotherapy response (Fig. 2b). The final gene panel included 4 downregulated (i.e. GNG11, IGFBP7, PLCG2 and SLC15A3) and  6 upregulated (i.e. CKB, CTNNBL1, RNF24, TSPAN31, TTI1 and UQCC1) genes (Table 2). The random forest classification model based on the ten-gene signature accurately differentiated S and R patients. Specifically, the prediction performance of Random Forest classifier, using the ten-gene expression data, showed classification accuracy of about 93% and precision of 94% (Table 3). These significant results highlight the importance of considering multigene interactions for the generation of a signature, which may be achieved through machine-learning pattern recognition.

**Table 2:** Ranking using Random Forest Classifier (RFC) of DEGs identified by Wilcoxon test

Gene symbol	MDA	MDG	MDA (%)	MDG (%)	Mean Percent	Wilcoxon *P*-value	Estimated shift	95% Conf. Interval
GNG11	20.13	2.25	96.49	100.00	98.25	0.0048	−1.09	−1.71, −0.34
SLC15A3	20.70	1.55	100.00	48.86	74.43	0.0293	−0.98	−1.75, − 0.17
PLCG2	20.24	1.57	97.16	50.18	73.67	0.0259	−0.91	−1.70, − 0.13
IGFBP7	13.04	1.50	52.42	45.17	48.80	0.0284	−0.42	− 0.89, − 0.06
CKB	12.57	1.46	49.47	41.73	45.60	0.0404	0.94	0.05, 1.79
RNF24	8.81	1.61	26.12	53.02	39.57	0.0050	0.59	0.20, 1.12
CTNNBL1	9.07	1.47	27.75	43.11	35.43	0.0178	0.53	0.05, 1.03
UQCC1	9.65	1.41	31.33	38.07	34.70	0.0101	0.54	0.15, 0.93
TSPAN31	4.60	1.51	0.00	45.95	22.97	0.0404	0.53	0.03, 0.98
TTI1	5.72	0.89	6.94	0.00	3.47	0.0275	0.59	0.06, 1.21

*DEGs* Differentially expressed genes, *MDA* Mean Decrease Accuracy, *MDG* Mean Decrease Gini impurity

**Table 3:** Random Forest Classifier (RFC) performances of 10-gene signature

Output	10 DEGs^a^
True Positive	15
True Negative	24
False Positive	1
False negative	2
Sensitivity	88.24
Specificity	96.00
Precision	93.75
F1	90.91
Accuracy	92.86
False Positive Rate	0.0400
False Discovery Rate	0.0625
False Negative Rate	0.0513

^a^Two subjects were excluded, given their Brier score > 1

### Epithelial markers of sensitivity to chemotherapy

**Fig. 3:**
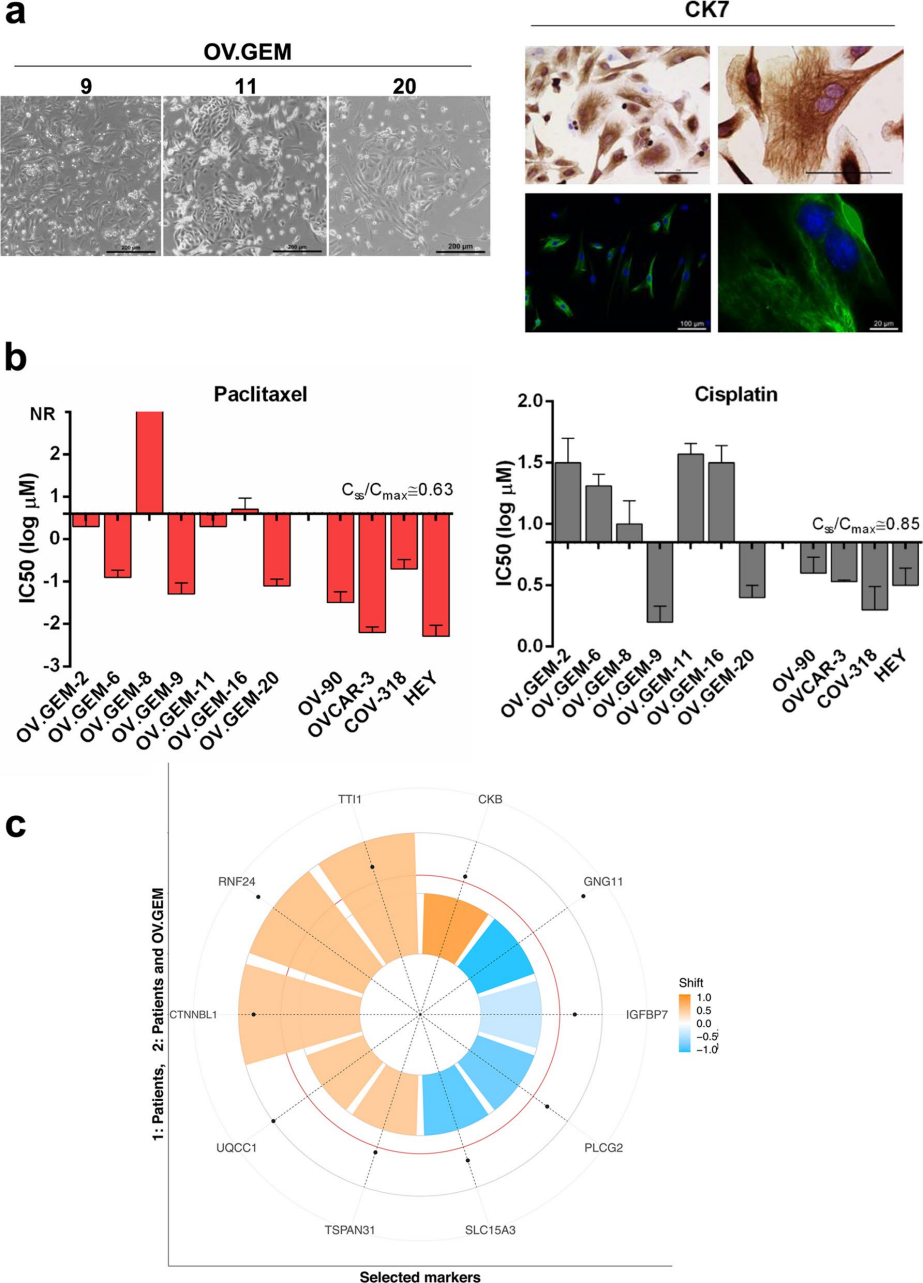
**a** Morphology of primary HGSOC cell lines (scale bars 200 μm) and representative images of cytokeratin 7 immunohistochemical and immunofluorescence staining (scale bars 100 and 20 μm). **b** Waterfall plot for paclitaxel and cisplatin IC_50_ values (extracted from dose-response curves) of primary and established HGSOC cell lines. The steady state (Css) or the maximum in vivo plasma concentrations are indicated by the solid line (see Table S5). Bars above the solid line represent the resistant samples and bars below represent the sensitive samples. **c**) Polar bar plot of the 10 selected biomarkers. Ten biomarkers have been chosen from the intersection of the top-42 RNA-seq DEGs with BH-adjusted P-value less than 5E-03 and |log2FC| > 1 and HT RT-qPCR two-sided Wilcoxon rank sum test *P* value < 0.05. For each of them, the bar colour shading corresponds to the estimated resistant – sensitive shift, and the bar height corresponds to the number of evidences supporting their regulation (1: patients only; 2: patients and OV.GEM cell lines). The black dots show the -log10 of the Wilcoxon’s P-value (the further the distance from the center, the stronger the significance). The red circle corresponds to a *P* value of 0.05

It is well known that tumor microenvironment may considerably shape responses to drug treatment, a condition that could partially explain discrepancies between drug sensitivity in vitro and in vivo [34]. In the light of this, results from transcriptomic analysis on surgical resections might have captured also molecular features of context-dependent therapeutic resistance. Therefore, we used in vitro cellular models to reveal, among the predictive biomarkers identified, those genes possibly involved in cell-autonomous mechanisms of resistance [i.e. independent from the tumor microenvironment (TME)]. To this end, ex vivo cultures from tumor tissues of treatment-naıve patients were established. Before testing, cellular morphology and cell-type protein expression analysis were assessed to determine the purity of cells following isolation and culturing. Selected cell lines had typical primary epithelial culture morphology and their epithelial nature was confirmed by cytokeratin 7 staining (Fig. 3a). The absence of stromal fibroblast overgrowth in the primary cell lines generated was verified by TE-7 staining (Fig. S2a).

We then evaluated the cytotoxicity of cisplatin and paclitaxel on a panel of seven different primary cell lines and calculated the half maximal inhibitory concentration (IC50) for each drug/cell line (Table S5). Cells were classified as resistant to either cisplatin or paclitaxel when the IC50 value was higher than the concentration achievable in patient plasma (concentration steady state/maximum concentration, i.e. Css/Cmax), and as sensitive when the IC50 value was lower than Css/Cmax, as previously reported [35, 36]. Results obtained showed that two out of the seven primary cell lines were resistant to both drugs (i.e. OV.GEM-8 and OV.GEM-16) (Fig. 3b, Table S5). Notably, at least for OV.GEM-8, drug response of the cell line correlated with patient outcome (Table S1). For the purpose of our analysis, OV.GEM-8 and OV.GEM-16 were thus considered as resistant models, while the remaining five primary cell lines as sensitive models. We next evaluated in these cell lines the expression of the ten DEGs identified as associated to drug response in patients. Interestingly, as depicted in the Polar chart, when considering the specific gene deregulation associated to drug sensitivity, CTNNBL1, RNF24 and TTI1 showed in primary HGSOC cell lines the same trend observed in tumor tissue from HGSOC patients (Fig. 3c). Data obtained give support to the hypothesis that these three genes, may represent tumor cell-derived resistance factors.

We also tested, for comparison, the cytotoxicity of cisplatin and paclitaxel on a panel of established cell lines, selected among those considered as really representative of HGSOC [15, 37]. Representative pictures illustrating cell morphology and cytokeratin 7 staining of the tested cell lines are shown in Fig. S2b. Notably, all the investigated established cell lines showed IC50 values below the Css/Cmax and therefore no one could be identified as a resistant model according to the above specified criteria (Fig. 3b, Table S5). Our results confirm the low translational relevance of established cancer cell lines for the identification of clinical anti-cancer drug resistance [38].

## Discussion

Here we have identified a ten-gene signature predictive of response to first-line chemotherapy in BRCAwt HGSOC patients. The signature includes downregulated (i.e. GNG11, IGFBP7, PLCG2, and SLC15A3) and upregulated (i.e. CKB, CTNNBL1, RNF24, TSPAN31, TTI1 and UQCC1) genes. Importantly, our classification model achieved very high accuracy and precision, demonstrating its potential use as actionable clinical indicator.

**Fig. 4:**
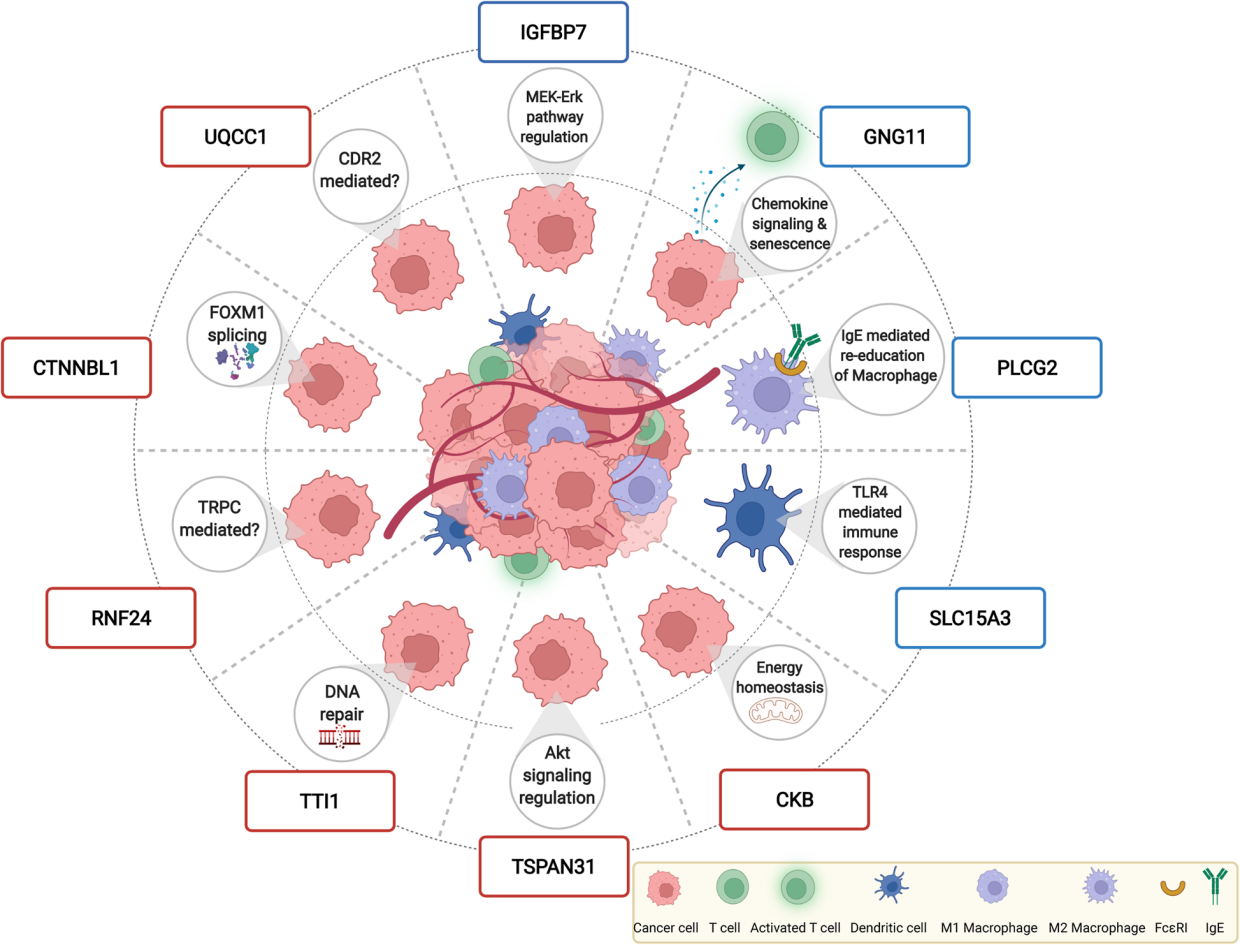
Picture showing the potential role of the identified genes in the development of cell-autonomous and non–cell-autonomous resistance to antineoplastic agents, according to literature data. Created with BioRender.com

Overall results obtained from the present study suggest that both cell-autonomous and TME-driven non-cell-autonomous mechanisms of drug resistance are critical in causing refractoriness of BRCAwt HGSOC patients and antineoplastic treatment failure (Fig. 4). Specifically, only three out of the ten DEGs identified in tumor biopsies were found to be similarly deregulated in primary HGSOC cells, i.e. CTNNBL1, RNF24 and TTI1, this implicating a role for these genes in tumor cell exclusive pathways of chemotherapy resistance. For the remaining biomarkers identified, as discussed in detail below for each of them, our findings support a role in different signaling pathways possibly driven by interactions between tumor cells and components of the extracellular matrix. These findings are in line with recent evidences indicating that chemoresistance in HGSOC stems from complex, and not fully understood, mechanisms linked to pre-existing gene expression intrinsic within the chemotherapy-naive tumor cells or the immediate microenvironment (revised in 6, [39]). In this context it is worthy to note that the pre-existing state of the tumor immune microenvironment has been demonstrated to play a key role in inherent-drug resistance in HGSOCs, infiltrated by a massive amounts of innate and adaptive immune cells [40].

Among the pathways we identified as significantly dysregulated in resistant patients, the Fc epsilon RI signaling (FcεRI) is of particular interest with regard to the TME-driven non-cell-autonomous resistance to antineoplastic agents. Indeed, recent fascinating hypotheses give support to the notion that, in tumor, IgE via interaction with its receptors (FcεRI and CD23) can engage and re-educate alternatively-activated macrophages (TAMs) towards pro-inflammatory phenotypes and prime all subsets to mediate anti-tumor functions, finally pointing to IgE as a novel anti-cancer modality [41–43]. Noteworthy, TAMs, the most abundant population of tumor-infiltrating immune cells in TME, with high density in HGSOC [44, 45], are critical in fostering tumor progression and chemoresistance in ovarian cancer [46]. KEGG analysis revealed that the FcεRI pathway was enriched in downregulated genes including PLCG2, ALOX5AP and INPP5D. In particular, PLCG2, one of the gene in our signature, is a critical signaling molecule in both the innate and adaptive immune systems [47], being activated in monocytes, macrophages, and mast cells through crosslinking of the FcεR and FcγR [48]. Since macrophages actually represent the major FcεR-expressing effector cells, we could speculate that PLCG2 deficiency, by impairing the FcεR signaling in macrophages, will finally reduce the proinflammatory and anti-tumoral states induced by IgE, with a strong impact on response to therapy. Notably, bioinformatic analysis of publicly available ovarian cancer transcriptome datasets corroborates our hypothesis, by showing a favorable prognostic role of PLCG2 expression in advanced-stage HGSOC (data not shown).

Consistent with a role of the immune system in driving chemosensitivity are also our findings showing a downregulation of SLC15A3 in R vs S patients. Indeed, SLC15A3 is a peptide/histidine transporter preferentially expressed in the immune cells, especially those of the innate immune system [49]. Despite the limited information available, SLC15A3 has been reported to be regulated by various Toll-like receptors (TLR), playing an important role in regulating TLR4-mediated inflammatory responses [50]. Relevant to our results, previous studies have demonstrated that TLR4-mediated immune response to dying tumor cells is essential for the efficacy of anticancer chemotherapy and radiotherapy; specifically, it has been reported that in the treatment of established tumors with chemotherapy or radiotherapy the presence of TLR4 dictates the therapeutic outcome [51].

A dual role in mediating chemotherapy resistance is suggested by literature and current data for GNG11, a member of the heterotrimeric guanine nucleotide-binding protein (G protein) gamma family complex, playing a vital role in the transmembrane signaling system. GNG11 was the top down regulated gene in our analysis, and although very little is known about this gene, it has been reported to work as a novel type of regulator in cellular senescence following oxidative stress [52]. Crucially to our findings, incomplete and heterogeneous senescence response of cancer cells contributes to chemotherapy resistance [53]. Besides, in the KEGG pathway analysis, GNG11 was found enriched in chemokine signaling pathway (along with PLCG2, PLCB1 and CXCL16), and studies have uncovered the importance of G protein-coupled receptor (GPCR) for the maintenance of T cell trophic state, growth, and proliferation [54]. This is particularly significant when considering evidence indicating that the antitumor activity of cisplatin is not limited to its ability to inhibit mitosis, but it also has important immunomodulatory effects, including recruitment and proliferation of CD8+ T cells [55]. On the whole, therefore, findings available suggest that GNG11 could be involved in different signaling pathways within cancer cells and TME.

Finally, among the downregulated genes, IGFBP7 (also known as Mac25 or IGFBP-related protein 1) encodes a secreted IGFBP-related protein, with binding affinity to insulin growth factor (IGF). IGFBP7 also possesses an IGF-independent activity [56]. Notably, it has been proposed as a candidate tumor suppressor gene in several cancer types, including ovarian cancer [57], although conflicting results in different tumors suggest that it IGFBP7 functions may be a “double-edged sword” in cancer proliferation, progression, and prognosis. Relevant to our results, loss of IGFBP7 has been associated with chemo-resistance to cisplatin in human cancer cell lines and lung xenografts, possibly by the extracellular signal-regulated kinase (ERK) mitogen-activated protein kinase (MEK-ERK) pathway regulation [58].

Although limited information is available with regard to their specific function, the six upregulated genes in the signature (CKB, CTNNBL1, TSPAN31, TTI1, RNF24 and UQCC1) have all been previously reported to be involved in the development of cancer and/or in mediating resistance to drug-induced cytotoxicity. In detail, an interesting correlate of drug response captured by our study was represented by KIAA0406/TTI1. Indeed, this protein has been identified as a critical component of DNA damage response (DDR), required for DNA damage resistance and whose depletion leads to cellular sensitivity to radiation, as well as other genotoxic agents [59]. Results from the present study are fully coherent with this proposed function.

With regard to CTNNBL1, UQCC1 and CKB, all of them have been previously associated to ovarian cancer. Specifically, the spliceosome-associated factor CTNNBL1, has been related to proliferation and invasion in HGSOC, at least partially through regulation of FOXM1 splicing [60], Notably, the FOXM1 signaling pathway is activated in 84% high-grade serous ovarian cancer [61] and linked to platinum resistance [62, 63]. Likewise, UQCC1, a protein involved in cytochrome b translation and/or stability, has been recently identified as a candidate ovarian cancer susceptibility gene [64]. Interestingly, it has been reported that UQCC1 physically interacts with CDR2 [65], a target antigen of naturally occurring human tumor immunity, widely expressed in the majority of ovarian cancer. Relevant to our results, recent studies have underscored the importance of CDR2 as target of an effective immune response in patients with paraneoplastic cerebellar degeneration (usually observed in gynecological cancer), pointing to its role in fueling immunogenic cell death (ICD) initiated by some chemotherapeutic agents [33, 66]. The inverse correlation between UQCC1 and CDR2 expression in ovarian cancer (as highlighted by analysis of TCGA data), gives support to our results. Finally, CKB gene expression has been reported to be up-regulated in ovarian cancer cells in vitro and in vivo and CKB enzyme activity to be significantly elevated in sera from ovarian cancer patients, including those with stage I disease [67]. Mechanistic studies have suggested that CKB is involved in the regulation of energy homeostasis and metabolic state in ovarian cancer [68].

Very little is known about the remaining two up-regulated genes in our analysis, i.e. TSPAN31 and RNF24. TSPAN31, recently discovered to be linked to cancer, as natural antisense transcript of cyclin dependent kinase 4 (CDK4), regulates the expression of CDK4 mRNA and protein [69]. Notably, TSPAN31 is also involved in the activation of Akt signaling pathway, a key regulator of survival in ovarian cancer, also associated with chemoresistance [70]. Lastly, RNF24 is a membrane protein which interacts with TRPC (transient receptor potential channel) proteins [71] and, although literature data have suggested that it may act as a cancer-promoting factor in different tumors [72, 73], its mechanistic role is still to be uncovered.

Besides patients’ stratification, findings from the present study could also aid to define potential new treatment strategies. Here we used primary HGSOC cell lines as a faithful experimental model of disease to identify, among the panel of the ten predictive biomarker genes, those candidates that we could investigate in in vitro experimental system, due to their possible cell-autonomous mechanisms of resistance. Interestingly, we showed that CTNNBL1, RNF24 and TTI1 are associated to resistance to therapy in both primary HGSOC cell lines and patients’ samples, in line with available literature data on the role of these proteins as tumor cell-derived resistance factors. On the other hand, established HGSOC cell lines, although easier to work with than primary cells, being genetically modified/transformed, may not fully represent the in vivo state and, therefore, may not be recommended for the identification of clinically relevant mechanisms of resistance to cytotoxic and targeted anti-cancer drugs.

Among the peculiar strengths of this study is that patients were treated in a single tertiary-care center, with accurately completed clinical records. In addition, all samples were managed and analyzed with standard protocols. Finally, we employed rigorous standardized normalization and statistical selection to overcome confounding factors and discrepancies resulting from differences in the experimental groups. Besides the above-mentioned strengths, few limitations in our study need to be acknowledged. First, the retrospective nature of this study makes it susceptible to bias. In addition, sample size was not large enough for detecting definitive associations between biomarkers expression and response to therapy. Therefore, validation of the gene signature in prospective trials is warranted. Beside, future studies are needed to pinpoint the mechanisms underpinning the role of the identified genes in drug-resistance and to investigate their potential druggability.

## Conclusions

Predicting platinum sensitivity still remains a challenge in women affected by HGSOC. Our results show that the Random Forest Classification model based on the expression levels of a limited number of marker genes could accurately predict response to first-line chemotherapy in BRCAwt patients. Further validation of this profile will contribute to establishing auxiliary predictive markers for the therapy of HGSOC, defining better and more effective clinical treatments, finally helping the field moving toward a more personalized treatment approach.

## Supplementary Information

**Additional file 1: Figure S1.** Pathology-based estimates of the percentage of tumor cells were evaluated from hematoxylin-eosin-stained slides. Only samples with higher than 70% of tumor purity were analyzed. **Figure S2.** a) Representative pictures of primary HGSOC cells stained using cytokeratin 7 (CK7) and TE-7 antibodies. The negligible staining for TE-7 (arrowhead) confirmed the absence of stromal fibroblast overgrowth in the primary cell lines generated. b) Morphology of established HGSOC cell lines and representative images of CK7 immunohistochemical staining. **Table S1.** Clinicopathological features of HGSOCs used for primary cell line isolation. **Table S2.** List of primers DeltaGene (Fluidigm) used in RT-qPCR. **Table S4.** Top DEGs in resistant vs sensitive HGSOC patients (downregulated genes). Top DEGs in resistant vs sensitive HGSOC patients (upregulated genes). **Table S5.** IC50 values of anticancer drugs against primary and commercially available HGSOC cell lines.

**Additional file 2: Table S3.** Containing RNA-seq raw data.

## Data Availability

All data generated during this study are included in this published article [and its supplementary information files]. The datasets analysed during the current study are available in the CBioPortal repository (https://www.cbioportal.org/) (accessed on 15 September 2021) and GeneMANIA repository (https://genemania.org).

## Abbreviations

ATCC: American Type Culture Collection
BH: Benjamini-Hochberg
BRCA1: Breast Cancer gene 1
BRCA2: Breast Cancer gene 2
BRCAwt: Breast Cancer gene 1/2 wild type
CDK4: Cyclin dependent kinase 4
cDNA: Complementary DNA
CDR2: Cerebellar degeneration-related protein 2
CKB: Creatine kinase B
Css/Cmax: Concentration steady state/maximum concentration
Ct: Cycle threshold
CTNNBL1: Catenin beta like 1
CV: Cross-validation
DAB: 3,3′-diaminobenzidine substrate
DAPI: 4′,6-diamidino-2-phenylindole
DDR: DNA damage response
DEGs: Differentially expressed genes
DMEM: Dulbeccoʼs modified Eagle′s medium
DNA: Deoxyribonucleic acid
ECACC: European Collection of Cell Cultures
ECM: Extracellular matrix
ERK: Extracellular signal-regulated kinase
FBS: Fetal bovine serum
FC: Fold changes
FDR: False Discovery Rate
FNR: False Negative Rate
FOXM1: Forkhead box M1
FPR: False Positive rate
G protein: Guanine nucleotide-binding protein
GAPDH: Glyceraldehyde-3-phosphate dehydrogenase
GCIG: Gynecologic Cancer Intergroup
GNG11: G protein subunit gamma 11
GPCR: G protein-coupled receptor
HGSOC: High-grade serous ovarian cancer
HRD: Homologous recombination deficiency
HT: High-throughput
IC50: Half maximal inhibitory concentration
ICD: Immunogenic cell death
IDS: Interval debulking surgery
IGFBP7: Insulin like growth factor binding protein 7
KEGG: Kyoto Encyclopedia of Genes and Genomes
MDA: Mean decrease accuracy
MDG: Mean decrease Gini
MEK-ERK: Mitogen-activated protein kinase
MEM: Minimum Essential Medium
NACT: Neoadjuvant chemotherapy
OD: Optical density
ORA: Over Representation Analysis
OV.GEM: Anonymous numerical codes for primary cell lines
padj: Adjusted *P* value
PARP: Poly (ADP-ribose) polymerase
PARPI: PARP inhibitors
PBS: Phosphate buffered saline
PDS: Primary cytoreductive surgery
PFI: Platinum-free interval
PLCG2: Phospholipase C gamma 2
PPV: Positive Predictive Value
R: Resistant
RECIST: Response Evaluation Criteria in Solid Tumors
RFC: Random Forest Classifier
RICF: Residual Iterative Conditional Fitting
RNA: Ribonucleic acid
RNA-Seq: RNA sequencing
RNF24: Ring finger protein 24
RPL13A: Ribosomal protein L13a
RPL4: Ribosomal protein L4;
RPMI: Roswell Park Memorial Institute Medium
RT: Room temperature
RT-qPCR: Quantitative real-time Polymerase chain reaction
S: Sensitive
SD: Standard deviation
SLC15A3: Solute carrier family 15 member 3
STR: Short Tandem Repeat
TAMs: Tumor-associated macrophages
TGCA: The Cancer Genome Atlas
TLR: Toll-like receptors
TMAP: Torrent Mapping Alignment Program
TME: Tumor Microenvironment
TRPC: Transient receptor potential channel
TSPAN31: Tetraspanin 31
TTI1: TELO2 interacting protein 1
UQCC1: Ubiquinol-cytochrome c reductase complex assembly factor 1
WT: Wild type
