# Supplementary material for: Identification of a novel gene signature predicting response to first-line chemotherapy in BRCA wild-type high-grade serous ovarian cancer patients
Source: J Exp Clin Cancer Res. 2022 Feb 4;41:50. doi: 10.1186/s13046-022-02265-w (PMC8815250; doi:10.1186/s13046-022-02265-w)
Supplement: Supplementary file 1 — Additional file 1: Figure S1. Pathology-based estimates of the percentage of tumor cells were evaluated from hematoxylin-eosin-stained slides. Only samples with higher than 70% of tumor purity were analyzed. Figure S2. a) Representative pictures of primary HGSOC cells stained using cytokeratin 7 (CK7) and TE-7 antibodies. The negligible staining for TE-7 (arrowhead) confirmed the absence of stromal fibroblast overgrowth in the primary cell lines generated. b) Morphology of established HGSOC cell lines and representative images of CK7 immunohistochemical staining. Table S1. Clinicopathological features of HGSOCs used for primary cell line isolation. Table S2. List of primers DeltaGene (Fluidigm) used in RT-qPCR. Table S4. Top DEGs in resistant vs sensitive HGSOC patients (downregulated genes). Top DEGs in resistant vs sensitive HGSOC patients (upregulated genes). Table S5. IC50 values of anticancer drugs against primary and commercially available HGSOC cell lines. [file 13046_2022_2265_MOESM1_ESM.pdf]

SUPPLEMENTARY MATERIALS FOR

IDENTIFICATION OF A NOVEL GENE SIGNATURE PREDICTING RESPONSE TO FIRST-LINE CHEMOTHERAPY IN BRCA WILD-TYPE HIGH-GRADE SEROUS OVARIAN CANCER PATIENTS

Marianna Buttarelli, Alessandra Ciucci, Fernando Palluzzi, Giuseppina Raspaglio, Claudia Marchetti, Emanuele Perrone, Angelo Minucci, Luciano Giacò, Anna Fagotti, Giovanni Scambia, and Daniela Gallo

Correspondence to: Daniela.gallo@unicatt.it

This PDF file includes:

Fig. S1

Fig. S2

Tables S1, S2, S4, S5 (Table S3 containing RNA-seq raw data is supplied as excel file)

Figure S1

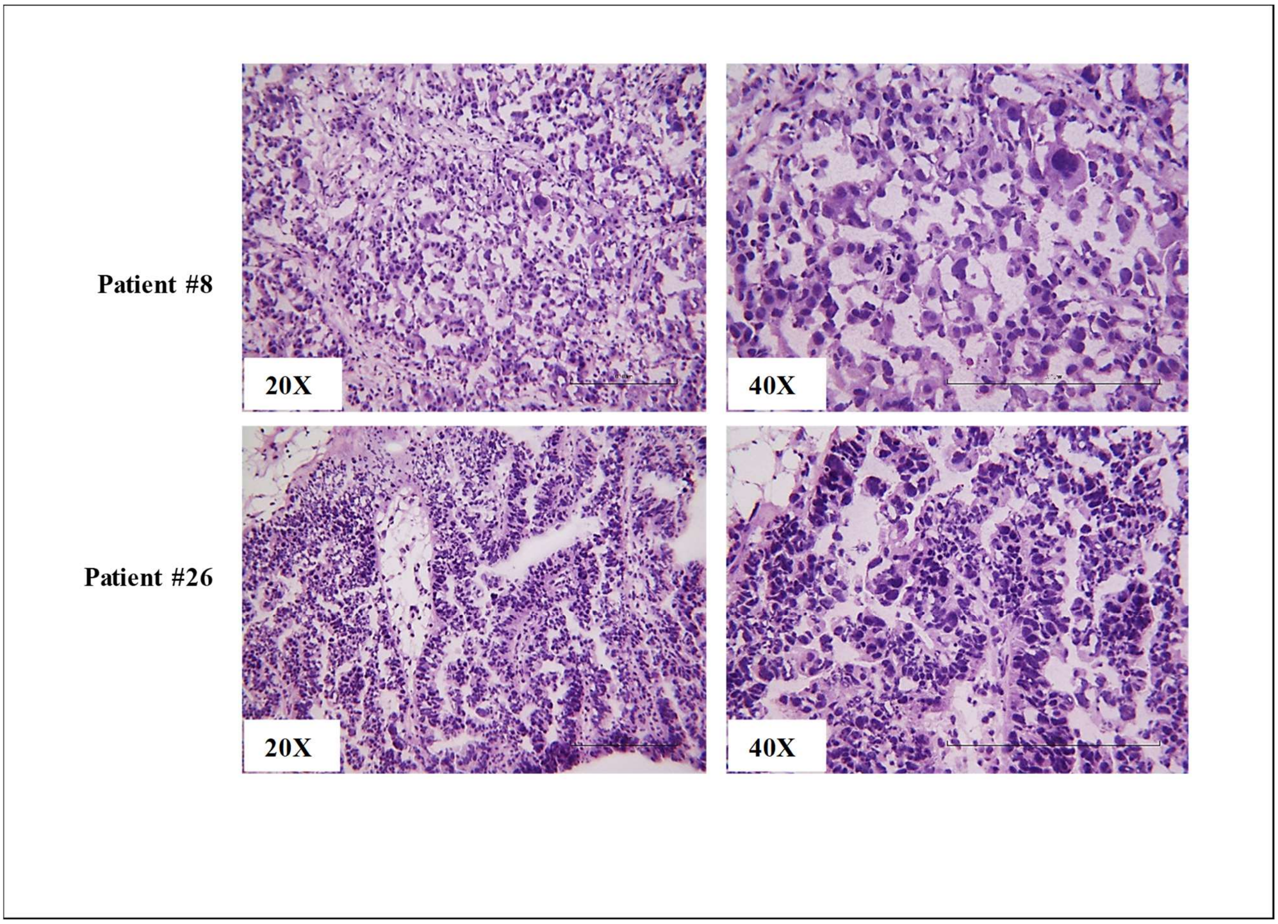

Figure S1. Pathology-based estimates of the percentage of tumor cells were evaluated from hematoxylin-eosin-stained slides. Only samples with higher than 70% of tumor purity were analyzed.

Figure S2

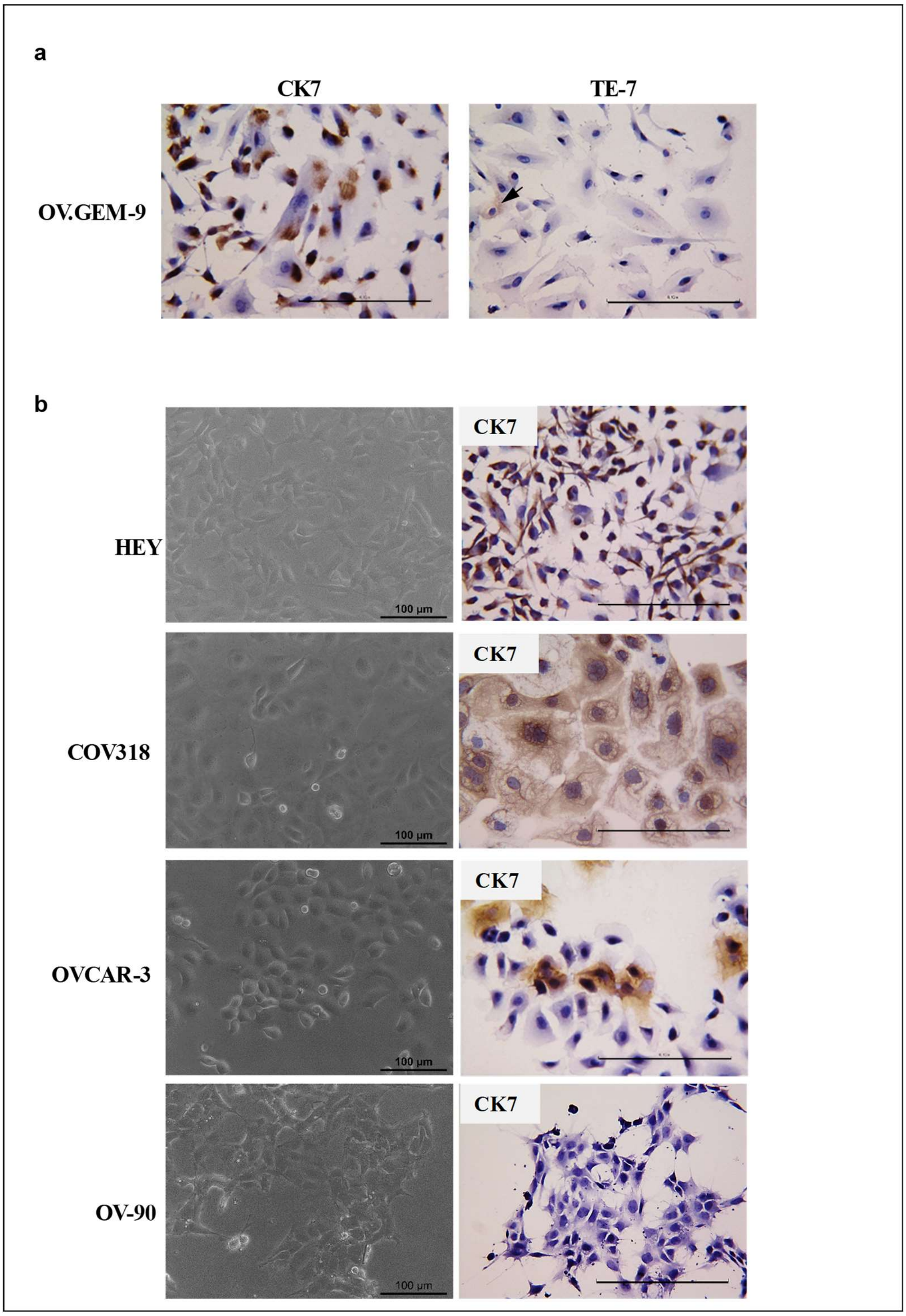

Figure S2. a) Representative pictures of primary HGSOC cells stained using cytokeratin 7 (CK7) and TE-7 antibodies. The negligible staining for TE-7 (arrowhead) confirmed the absence of stromal fibroblast overgrowth in the primary cell lines generated. **b)** Morphology of established HGSOC cell lines and representative images of CK7 immunohistochemical staining.

TABLE S1. Clinicopathological features of HGSOCs used for primary cell line isolation

Primary cell line	Age	Primary site	Figo Stage	Somatic BRCA	Germline BRCA	P53	CK7	PAX8	WT1	Primary Chemotherapy	Recurrence	PFI
**OV.GEM-2**	63	Ovary	IVB	WT	WT	Mut	+	+	+	Platinum/Paclitaxel Bevacizumab	No	> 12
**OV.GEM-6**	54	Omentum	IIIC	WT	WT	Mut	+	+	+	Platinum/Paclitaxel Niraparib	No	> 11
**OV.GEM-8**	69	Omentum	IIIC	WT	*na*	Null type	+	+	+	Platinum/Paclitaxel	Yes	1
**OV.GEM-9**	38	Ovary	IVB	WT	WT	Mut	*na*	*na*	*na*	Platinum/Paclitaxel Niraparib	No	> 11
**OV.GEM-11**	65	Ovary	IIIC	WT	WT	Mut	+	+	+	Platinum/Paclitaxel Niraparib	No	> 8
**OV.GEM-16**	53	Ovary	IVB	WT	*na*	Mut	+	+	+	Platinum/Paclitaxel Bevacizumab	No	> 6
**OV.GEM-20**	62	Peritoneum	IIIC	WT	*na*	Mut	+	+	+	Platinum/Paclitaxel Bevacizumab	No	> 4

HGSOC=high-grade serous ovarian cancer; PFI = Platinum free interval; *na*= not available

Table S2. List of primers DeltaGene (Fluidigm) used in RT-qPCR

Target gene	GENE ID	Forward primer (5'→3')	Reverse primer (5'→3')
**ABHD5**	51099	GATGTGCCCTAGGATTGGACAA	CAGCAAGGTCTGGTCGTTCA
**ACVR1B**	91	AGAAGCTGCGTCCCAACA	ACACTCTCGCATCATCTTCCC
**ALOX5AP**	241	TTCCAGAGGACCGGAACAC	GGAAAGTGGGGTACGCATCTA
**C3AR1**	719	TGACGATCAAGTGCCAACAC	ATAACAGAGGGCAGCAGGAA
**CD4**	920	ACCTGTACAGCTTCCCAGAA	GAGCCCTGATTTCCCAGAATC
**CKB**	1152	CTTCTGCACCGGCCTCA	TAGCCCAGGTGAGGGTTCC
**CPZ**	8532	TGCTGGTCATCGAGTTCTCC	GAATGTTGCCGATGAGCTTCA
**CTNNBL1**	56259	TGCTGGCCATATTGCTCCA	GAAGAAGCACATCGATTCCATCC
**CXCL16**	58191	TTTCCCACCCTCCCTCCTTA	GGACTGAGGAGGTGAAACCAA
**DHX35**	60625	AAGGAAGAGTGGTAGGAGTGAC	GCACCCCTTTCTTCAGCTAC
**DSN1**	79980	GCATCTGTGGCTGAGATGAA	AGTGAAGCAAGAGCTGATCC
**GAPDH**	2597	AGTCCACTGGCGTCTTCAC	TGATCTTGAGGCTGTTGTCATACTTC
**GNG11**	2791	TGAGCAGCTTCGCAAAGAA	AGGGGTTCTTGTCTTCTGGAA
**HERC5**	51191	TTCAGATCACATGTGGAGATTACC	GTTCTGTCCCCAGGCAAAA
**HSP90AB1**	3326	TCTCGCATGAAGGAGACACA	CGCACTCGCTCCACAAAA
**IGFBP7**	3490	GTGGCCCAGAAAAGCATGAA	GAATTGGATGCATGGCACTCA
**IGSF9**	57549	CCCCTACAACAGTCTTGGTAC	CCTTGGGCCGCTCTATAAAA
**INPP5D**	3635	CACATTTGAGGCAGGAGTCAC	TCAATCTGTCCTTGGCTGTCA
**LDLR**	3949	ACGAATTCCAGTGCTCTGATG	GCTCATGTCCTTGCAGTCAT
**LINC01816**	100133985	GGTTGGCGTGCTTGTACAC	TCCAAGTAGAGAAAACCGGGAAC
**MILR1**	284021	ACGTAAGACACACCTGGGAA	TTCATGGGCTTCTGTGATGC
**MYO5A**	4644	AAAAGGTCTTGGCCTCCAAC	CTTCCCAAAACGGCTGCTA
**NEXN**	91624	GGGAAATTGATGCAGCACTACA	TCAGCAGTGGAGCCATTCA
**NOL4L**	140688	GCCTGTGAGAGCGAGACAA	GCTGCTTGTCCAGGGCTA
**PLCB1**	23236	ACTTAAGCTGCAAGTCACTCCA	TCGCTTCCGATCTGCTGAAA
**PLCG2**	5336	CTGCAAACCAACCAGCAAAAC	TCAGCCTTCGTCTCCACAAA
**PODN**	127435	GCAGAACAACTACCTGACTGAC	CTGGACAGATCCAGGTACTCC
**RNF24**	11237	CCATGTAAGCACGCCTTCC	TGCACAGGGGACACACTTTA
**RPL13A**	23521	GAGGCCCCTACCACTTCC	GCCGTCAAACACCTTGAGAC
**RPL4**	6124	TTCGAGGTGGTGGGACTCA	TGCAAACATTCGGCCTCCA
**RPLP0**	6175	GCGACCTGGAAGTCCAAC	CACATTGTCTGCTCCCACAA
**RPRD1B**	58490	ACGACTACCCTGGCAGCTAC	CCCTGATGCGGCATTTTCCA
**SELPLG**	6404	CCTTGTCACTAAAGCAGAGAAGCC	GGTGTCCCACAGCTGCAA
**SERPINF1**	5176	CCGATGAGATCAGCATTCTCC	AGGGAAGTCTTTCTGGAGTCA
**SLC15A3**	51296	GCTGATTCTGGTCCCTCTGAA	TTCTGCAGAGCAGAGGGAAG
**STOM**	2040	ACTCTGGATGATGCCACTGA	GCTGCACAGGTAGTTTCACA
**TACC1**	6867	TCTCTCGCCCTGGATGCA	ACGTGGATGCCAGTTTCTCC
**TARBP2**	6895	CTGGAACACTGGGCTTTATGC	AAGGCTGATCGGGGTCTTG
**TMEM140**	55281	GCCAGCGAGAAGGAAGATTCA	TTGGGCCGGCCATCTCTA
**TNFSF13B**	10673	GGGATGAATTGAGTCTGGTGAC	GTTGGAGTTCATCTCCTTCTTCC
**TPP1**	1200	TCTCAACCCAAGGCTCTACC	CTCTTCATCCAGACAGGACTCA
**TRPM2**	7226	AGCAACAGCAGCCTCTTCAA	TGATGTTTTCAGGAATCCACGAAC
**TSPAN31**	6302	TCAACCTCACAACCCTGTATCA	TTTTCAGGGCTTCGTCTGAA
**TTI1**	9675	AGAGAGCTGCTTCCTTTGTCA	GATTACCTGTGTCTGGGAACCA
**QCC1**	55245	CCACAGCCTGTTGAGGAGAA	TCAAAGGTCCCGTGAATCCC
**WDPCP**	51057	CATGAGCGCCATTGTAAACC	GGTTCCAAGGCTTGTCTCAA
**ZNF738**	148203	TGTGCGTATCTTTCCCAGAA	GGTGCTGAAAACAGTTGCT

Table S4. Top DEGs in resistant *vs* sensitive HGSOC patients (*downregulated genes*) - continues

Gene	Official name§	log_2FC	Molecular function*
**PODN**	Podocan	-2.51	Collagen binding
**CPZ**	Carboxypeptidase Z	-2.26	Carboxypeptidase, Hydrolase, Metalloprotease, Protease
**NEXN**	Nexilin F-actin binding protein	-2.15	Actin-binding
**SERPINF1**	Serpin family F member 1	-1.89	Serine-type endopeptidase inhibitor activity
**IGFBP7**	Insulin like growth factor binding protein 7	-1.88	Growth factor binding
**ALOX5AP**	Arachidonate 5-lipoxygenase-activating protein	-1.84	Arachidonic acid binding, Protein binding
**TRPM2**	Transient receptor potential cation channel, subfamily M, member 2	-1.81	Calcium channel, Ion channel, Sodium channel
**MILR1**	Mast cell immunoglobulin like receptor 1	-1.74	Transmembrane signaling receptor activity, Protein binding
**SELPLG**	Selectin P ligand	-1.69	Host cell receptor for virus entry, Receptor
**TMEM140**	Transmembrane protein 140	-1.62	Protein binding
**PLCG2**	Phospholipase C, gamma 2	-1.60	Hydrolase, Transducer
**C3AR1**	Complement component 3a receptor 1	-1.57	G-protein coupled receptor, Receptor, Transducer
**GNG11**	G protein subunit gamma 11	-1.52	Transducer
**TNFSF13B**	TNF superfamily member 13b	-1.52	Cytokine
**CD4**	CD4 molecule	-1.51	Host cell receptor for virus entry, Receptor
**INPP5D**	Inositol polyphosphate-5-phosphatase D	-1.42	Hydrolase
**TACC1**	Transforming, acidic coiled-coil containing protein 1	-1.41	Activator
**MYO5A**	Myosin VA	-1.27	Actin-binding, Calmodulin-binding, Motor protein, Myosin
**SLC15A3**	Solute carrier family 15 member 3	-1.23	Dipeptide transmembrane transporter activity
**LDLR**	Low density lipoprotein receptor	-1.17	Host cell receptor for virus entry, Receptor
**CXCL16**	C-X-C motif chemokine ligand 16	-1.15	Cytokine
**HERC5**	HECT and RLD domain containing E3 ubiquitin protein ligase 5	-1.09	Transferase
**STOM**	Stomatin	-1.06	Identical protein binding
**ABHD5**	Abhydrolase domain containing 5, lysophosphatidic acid acyltransferase	-1.03	Acyltransferase, Transferase
**TPP1**	Tripeptidyl peptidase 1	-1.03	Hydrolase, Protease, Serine protease

Table S4. Top DEGs in resistant vs sensitive HGSOC patients (*upregulated genes*)

Gene	Official name§	log_2FC	Molecular function*
**LOC100133985/ LINC01816**	Long intergenic non-protein coding RNA 1816	1.95	-
**TARBP2**	TARBP2 subunit of RISC loading complex	1.44	RNA-binding
**WDPCP**	WD repeat containing planar cell polarity effector	1.39	-
**ZNF738**	Zinc finger protein 738	1.31	DNA binding
**CKB**	Creatine kinase B	1.30	ATP binding, Kinase, Transferase
**IGSF9**	Immunoglobulin superfamily member 9	1.25	Developmental protein
**C20orf112/ NOL4L**	Nucleolar protein 4 like	1.20	RNA binding
**RNF24**	Ring finger protein 24	1.19	Protein binding
**UQCC1**	Ubiquinol-cytochrome c reductase complex assembly factor 1	1.17	Protein binding
**PLCB1**	Phospholipase C beta 1	1.15	Hydrolase, Transducer, Protein binding
**DHX35**	DEAH-box helicase 35	1.12	Helicase, Hydrolase
**RPRD1B**	Regulation of nuclear pre-mRNA domain containing 1B	1.12	Identical protein binding
**DSN1**	DSN1 component of MIS12 kinetochore complex	1.1	Protein binding
**CTNNBL1**	Catenin beta like 1	1.1	Enzyme binding
**TSPAN31**	Tetraspanin 31	1.05	Protein binding
**ACVR1B**	Activin A receptor, type IB	1.04	Kinase, Receptor, Serine/threonine-protein kinase, Transferase
**TTI1**	TELO2 interacting protein 1	1.03	Protein binding

DEGs = differentially expressed genes; HGSOC=high-grade serous ovarian cancer; FC = fold change; §source = NCBI gene; *sources = UNIPROT, neXtProt, PhosphoSitePlus®

Table S5. IC50 values of anticancer drugs against primary and commercially available HGSOC cell lines

**Cell line**	**IC50 (µM)**	
	**Paclitaxel**	**Cisplatin**
**OV.GEM-2**	1.9±1.3	34.8±16
**OV.GEM-6**	0.13±0.05	20.4±4.6
**OV.GEM-8**	>10	11.2±5
**OV.GEM-9**	0.05±0.03	1.7±0.5
**OV.GEM-11**	2.0±1.3	37.2±7.5
**OV.GEM-16**	5.5±3.3	35.2±11
**OV.GEM-20**	0.08±0.03	2.3±0.54
**COV-318**	0.2±0.1	1.8±0.8
**HEY**	0.005±0.003	3.1±1.0
**OV-90**	0.03±0.018	4.0±1.2
**OVCAR-3**	0.006±0.0018	3.4±0.1
**Css/Cmax**[§]	4.3[a]	7.4[b]

HGSOC=high-grade serous ovarian cancer; [§]Css = patient plasma concentration of drug at steady-state plateau; Cmax = patient maximum plasma concentration of drug; [a] de Witte *et al*. Cell Reports 2020;31:107762; [b] Nagai *et al*., Cancer Chemother Pharmacol 1996;39:131-7.
